# An analysis of the uptake of anti-retroviral treatment among pregnant women in Nigeria from 2015 to 2020

**DOI:** 10.1371/journal.pgph.0001749

**Published:** 2023-04-04

**Authors:** Akudo E. Ikpeazu, Samuel E. Anya, Richard N. Amenyah, Olugbenga A. Ijaodola, Adebobola T. Bashorun, Chinwendu Daniel Ndukwe, Abiola Davies, Mojisola Mobolaji-Bello, Chioma Ukanwa, Adaoha O. Anosike, Uduak Daniel, Doris A. Ogbang, Koubagnine V. Takpa, Olumuyiwa A. Ojo, Otse Ogorry, Murphy Akpu, Gregory Ashefor, Alex Ogundipe, Gambo G. Aliyu, Erasmus Morah

**Affiliations:** 1 National AIDS and STI Control Programme, Federal Ministry of Health, Abuja, Nigeria; 2 UNAIDS Country Office, Abuja, Nigeria; 3 National Agency for the Control of AIDS, Abuja, Nigeria; 4 United Nations Children’s Fund Country Office, Abuja, Nigeria; 5 World Health Organization Country Office, Abuja, Nigeria; 6 The President’s Emergency Plan for AIDS Relief Coordination Office, Abuja, Nigeria; Universidad de Chile, CHILE

## Abstract

The percentage of Human Immunodeficiency Virus (HIV) positive pregnant women that receive anti-retroviral treatment in Nigeria is low and has been declining. Consequently, 14% of all new infections among children in 2020 occurred in Nigeria. A detailed analysis of available data was undertaken to generate evidence to inform remedial actions. Data from routine service delivery, national surveys and models were analyzed for the six-year period from 2015 to 2020. Numbers and percentages were calculated for antenatal registrations, HIV testing, HIV positive pregnant women and HIV positive pregnant women on antiretroviral treatment. The Mann-Kendall Trend Test was used to determine the presence of time trends when the p-value was less than 0.05. In 2020, only 35% of an estimated 7.8 million pregnant women received antenatal care at a health facility that provided and reported PMTCT services. Within these facilities, the percentage of HIV-positive pregnant women on anti-retroviral treatment from 71% in 2015 to 88% in 2020. However, declining HIV positivity rates at these antenatal clinics and an absence of expansion of PMTCT services to other pregnant women due to cost-efficiency considerations contributed to a progressive decline in national PMTCT coverage rates. To achieve elimination of mother-to-child transmission of HIV, all pregnant women should be offered a HIV test, all who are HIV positive should be given anti-retroviral treatment, and all PMTCT services should be reported.

## Introduction

The elimination of mother-to-child transmission of HIV has been and remains a global priority since the first UN General Assembly Political Declaration on HIV and AIDS in 2006 [1–4]. The Start Free, Stay Free, AIDS Free framework was launched in 2015 in recognition of the need to accelerate progress towards ending the AIDS epidemic among children, adolescents and young women by 2020 [5]. The Start component of the framework focused on elimination of new HIV infections among children.

A recent review shows that progress has been slow with only a 20% decline in new infections among children globally by the end of 2020 compared to 80% targeted [5]. Among the 21 African countries that were prioritized in the Start Free, Stay Free, AIDS Free framework, only a 24% decline was achieved.

Nigeria, one of 21 African countries prioritized in the framework, accounted for 24% of pregnant women living with HIV who were not on treatment, 14% of children newly infected with HIV and 8% of AIDS-related deaths globally [6,7]. Although, there has been a 16% decline in children newly infected with HIV from 2010–2020, year-on-year analysis shows a 31% increase from 2014–2020 [8]. This is consistent with data showing that the percentage of pregnant women living with HIV on treatment increased from 31% in 2010 to peak at 72% in 2014 then declined progressively to 44% by end of 2020 [8]. Only two out of 17 countries in West Africa had a lower percentage of pregnant women living with HIV on treatment in 2020, namely Congo (14%) and Mali (28%) [7]. These make Nigeria the largest contributor to new HIV infections and AIDS-related deaths among children globally. A recent modes of transmission study showed mother-to-child-transmission was the second largest source of new HIV infections in the country accounting for 22% of all new HIV infections [9].

The high burden of HIV among children and declining treatment coverage among pregnant women are of great concern to government, development partners, civil society organizations, and networks of persons living with HIV. This led to a high-level national consultation in May 2021 chaired by the Honourable Minister of Health. The consultation highlighted the need for more detailed analysis to provide evidence for action to reverse the decline in the performance of Nigeria’s prevention of mother-to-child transmission of HIV (PMTCT) programme. This paper presents the findings of the detailed six-year analysis of national PMTCT coverage in Nigeria.

## Methods

### Approach

To understand the performance of Nigeria’s PMTCT Programme, the National AIDS and STI Control Programme (NASCP) in the Federal Ministry of Health set up a technical team to analyse all aspects of available data, decide what variables are within the control of the HIV programme and otherwise and to make suggestions for corrective action. The team focused on key components of HIV testing and treatment coverage for pregnant women living with HIV (numerator and denominator) with emphasis on how they are generated, reported and what critical questions should guide understanding of the data.

#### The PMTCT denominator

The denominator is a modeled estimate of the number of pregnant women living with HIV that is derived from assumptions including: population size from census projections; age-specific fertility rates for women in reproductive age from national demographic and health surveys (NDHS); HIV prevalence from the National AIDS Indicator and Impact Survey (NAIIS); and HIV positivity rates among pregnant women from routine programmatic data. The National Population Commission is responsible for census and census projections while the National Bureau of Statistics conducts demographic and health surveys. The Federal Ministry of Health had primary responsibility for the NAIIS and is responsible for programmatic data.

Apart from HIV positivity rates among pregnant women, these parameters are not subject to significant change based on actions of NASCP in the PMTCT programme. A change in the proportion of pregnant women tested may lead to a change in reported HIV positivity rates if rates among women at locations where testing is not done are significantly different from rates among women at locations where testing is done.

#### The PMTCT numerator

The numerator accounts for the number of pregnant women receiving antiretroviral therapy from service delivery points. These data are derived from the routine national programme service delivery dataset and are the focus of this analysis. It is dependent on a cascade involving identification of pregnant women, testing them, and providing anti-retroviral treatment to those who are HIV positive.

Based on this cascade, our analysis was guided by three questions related to the location where pregnant women receive antenatal care and delivery services, availability of services, and uptake of services leading to and including anti-retroviral treatment for pregnant women:

Q1. Where can pregnant women be found receiving antenatal care and delivery services? [Location]
Q2. Are HIV testing and treatment services provided at these locations? [Availability]
Q3. Do pregnant women accept to receive HIV testing and treatment services when available? [Uptake]

These questions were explored at national level for the period from 2015–2020 during which PMTCT services were delivered through health facilities.

### Sources of data

The analysis is based on a combination of modeled estimates, survey data, health facility registry and routine service delivery (programme) data. Modeled estimates provided data on the Estimated number of pregnant women living with HIV and estimated number of total births which was used as proxy for estimated number of pregnant women each year. These estimates served as denominators for calculating the coverages (the proportion of expected population who received a service—ANC, HIV testing and ARV). Survey data provided information on proportional distribution of women who received ANC or not and where such care was received. The proportion from the survey were applied to the estimated number of pregnant women to provide data on the estimated number of pregnant women who received skilled/facility-based ANC. The health facility registry provided information on the number of health facility that provides Antenatal care which was combined with the programme data to determine the proportion of health facilities providing PMTCT services. The programme data in combination with the above was used to establish programme performance and identify gaps in availability and access to services.

The data elements used for this analysis and their sources are shown in Table 1.

**Table 1:** Data used in analysis of ART coverage among pregnant women living with HIV and their sources.

	Data elements	Sources
1	Estimated number of pregnant women Estimated number of pregnant women living with HIV	UNAIDS Spectrum 2021, Nigeria file [8]
2	Percentage of pregnant women who received antenatal care by type of provider	Nigeria Demographic and Health Survey 2018 [10]
3	Number of health facilities providing antenatal care	Health Facility Registry, Federal Ministry of Health [11]
4	HIV prevalence among women and pregnant women	National AIDS Indicator and Impact Survey [12]
5	Data reported by each state using national reporting tool: Number of health facilities providing and reporting on HIV testing and treatment among pregnant women Number of pregnant women who received antenatal care from facilities reporting PMTCT Number of pregnant women tested for HIV Number of pregnant women who already knew their HIV status prior to antenatal care registration Number of newly diagnosed HIV positive pregnant women Number of pregnant women who already knew their status and were on ART at antenatal registration Number of newly diagnosed pregnant women who were on ART	Routine PMTCT Programme Data, National AIDS and STI Control Programme, Federal Ministry of Health (Table in SI Table)
6	Data from PEPFAR supported sites on: Number of pregnant women tested for HIV Number of positive pregnant women Number of positive pregnant women on ART Number of health facilities reporting on PMTCT services	PEPFAR Monitoring, Evaluation and Reporting Database [13]

### Analysis

For each year from 2015–2020, we prepared frequency tables on antenatal registrations, HIV testing, HIV positive pregnant women and HIV positive pregnant women on antiretroviral treatment.

Percentages were then computed for antenatal registrations and HIV testing among all expected pregnant women; HIV testing among women who registered for antenatal care; HIV positivity among women tested for HIV; and antiretroviral treatment among HIV positive pregnant women. The percentage of health facilities that provided antenatal care was also calculated. The Mann-Kendall Trend Test was used to determine the presence of time trends in the number of pregnant women seen and tested for HIV as well as the number of HIV positive pregnant women seen and the PMTCT coverage. If the p-value was less than 0.05, we concluded that a trend was present in the data. We checked for autocorrelation (serial correlation) for each variable before applying the Mann-Kendal Trend Test. There was no autocorrelation in the plots, therefore, the Mann-Kendall Test was used without variance correction.

The performance on HIV testing and anti-retroviral treatment at health facilities supported by PEPFAR was compared to performance at health facilities that were not supported by PEPFAR. Annual data for PEPFAR-supported facilities was reported from October of the previous year to September of the index year [13]. Quarterly data only became available from October 2016. Therefore, using 2016 as an example, Quarter 1 data was from October to December 2015 while Quarter 4 was from July to September 2016. To convert the data to January to December, we added Q2, Q3, Q4 of 2016 and Quarter 1 of 2017 (S4 Table). Quarterly data was not available for 2015 for any indicator and in 2016 for HIV positive pregnant women on ART, therefore, we did not include them in the analysis by funder. For number of reporting facilities in the index year, we used the number as reported because it was not possible to annualize from the available data.

The analysis was done using R version 4.1.2.[14].

### Ethical considerations

Ethical review was not required because the study involved the analysis of existing national programme and survey data that were publicly available.

## Results

### Where to find pregnant women receiving antenatal care and delivery services [Location]

The estimated number of pregnant women ranged from 6.9 million in 2015 to 7.8 million in 2020 [8]. The antenatal care coverage of 67% [10] means 4.6–5.2 million pregnant women were expected annually at the antenatal clinics in the country. Of the remaining 33%, 9% received antenatal care from unskilled providers while 24% did not receive any antenatal care.

**Fig 1:**
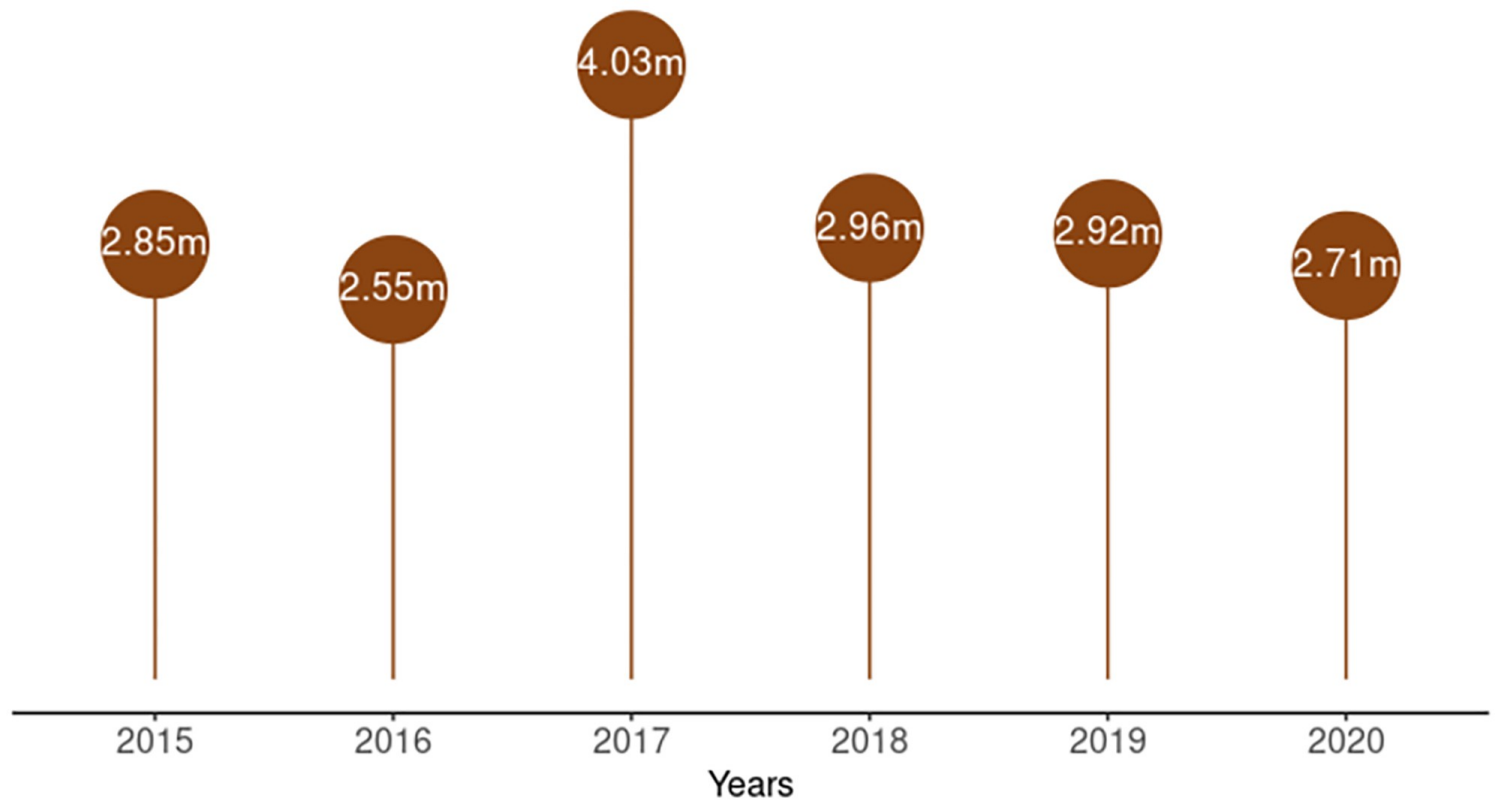
Lollipop chart of number of pregnant women seen at health facilities that report on PMTCT indicators from 2015–2020.

**Fig 2:**
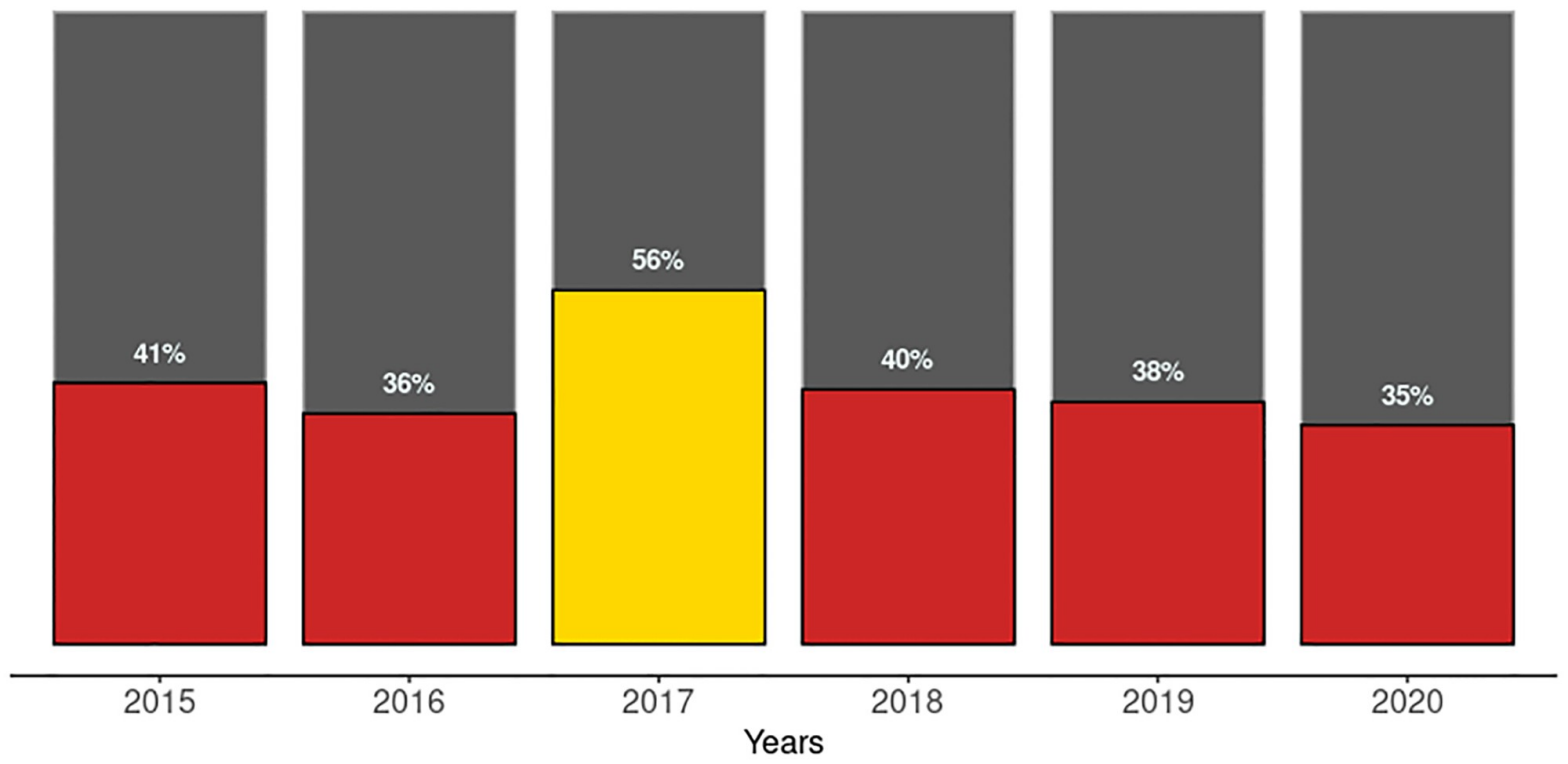
Battery chart showing trends in the number of pregnant women seen at PMTCT reporting facilities as a percentage of estimated pregnant women from 2015–2020.

During the period under review, 2.6–4.0 million pregnant women registered annually for antenatal care at health facilities that also provided and reported HIV testing and treatment services for pregnant women (Fig 1). The percentage of estimated pregnant women seen at PMTCT reporting facilities declined from 41% in 2015 to 35% in 2020, as shown in Fig 2. However, there was no trend during the period (tau = -0.0667, 2-sided p value = 1). In 2020, of the 5.2 million pregnant women expected to receive antenatal care from health facilities in the country, only 2.7 million (52%) received skilled antenatal care at PMTCT reporting facilities. The other 48% (2.5 million) received antenatal care at health facilities that did not provide PMTCT services or did not report the PMTCT services they provided.

### HIV testing and treatment services where pregnant women are located [Availability]

**Fig 3:**
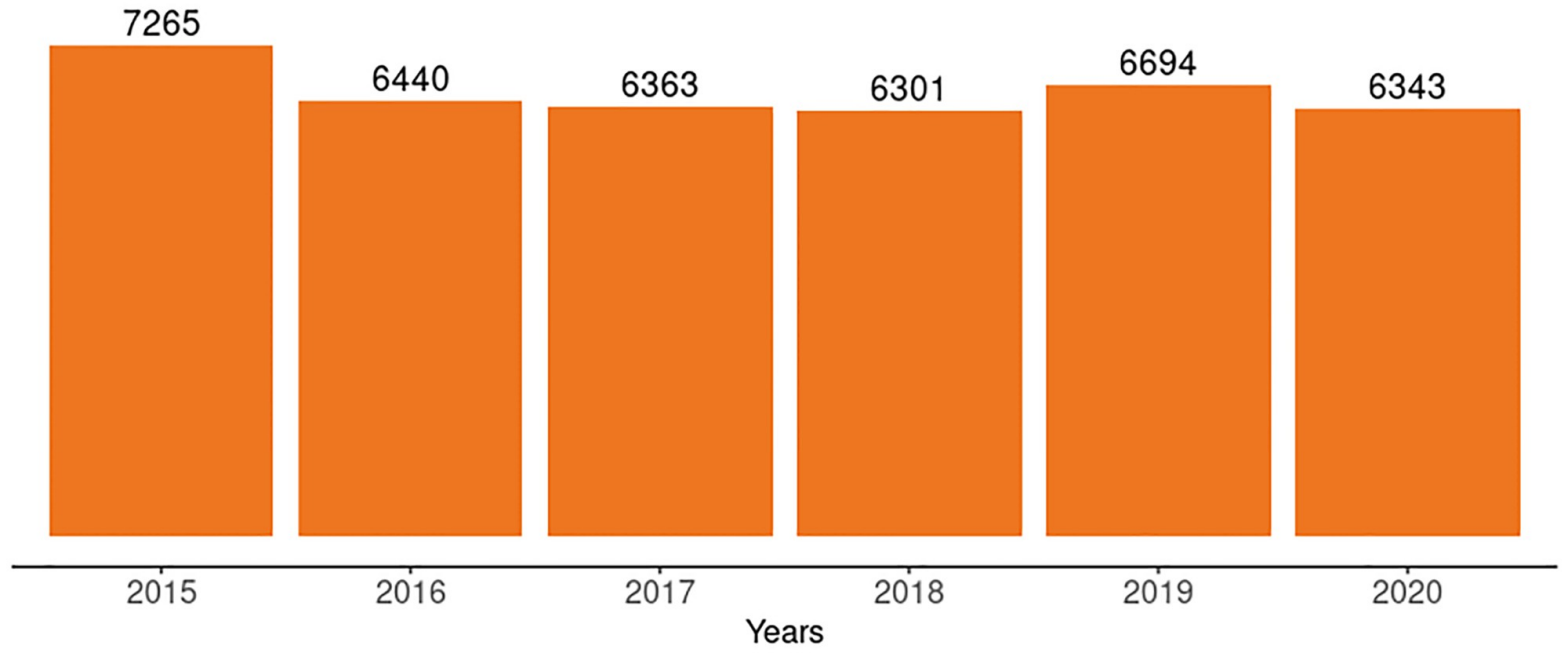
Number of health facilities that provided PMTCT services from 2015–2020.

During the period under review, PMTCT services were provided and reported through health facilities. The number of PMTCT reporting facilities declined by 12.7% from 7,265 in 2015 to 6,343 in 2020 (Fig 3). The total number of health facilities providing antenatal care is updated by the Federal Ministry of Health and in June 2021, there were 24,513 health facilities offering antenatal services which we used as a proxy for 2020. This showed that in 2020 25.9% of antenatal care facilities accounted for the 2.7 million pregnant women seen that year. For the other 2.5 million pregnant women expected to be seen at antenatal clinics in 2020, it is not known how many received antenatal care at health facilities that do not provide PMTCT services and how many received antenatal care at health facilities that provide but do not report PMTCT services.

**Fig 4:**
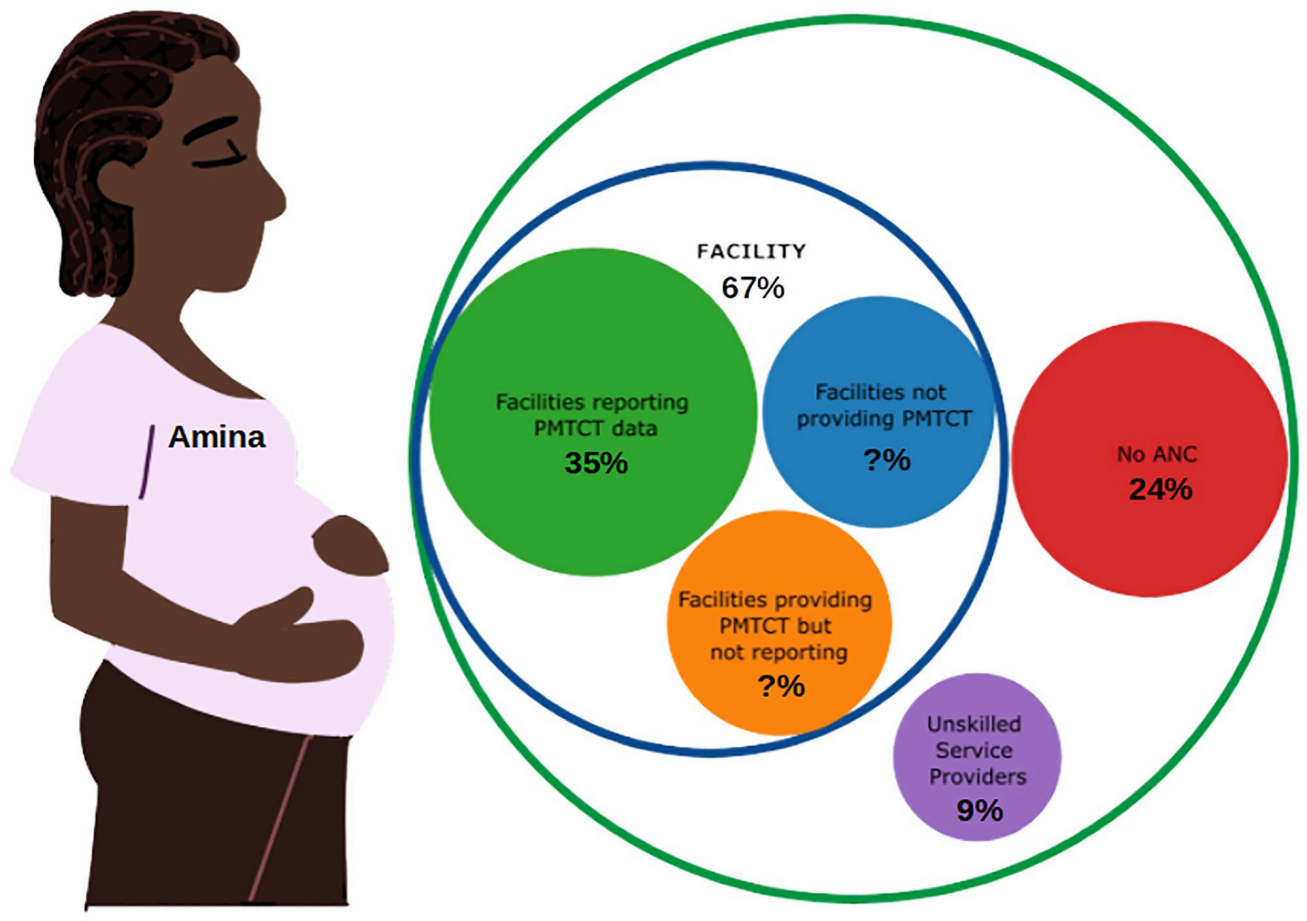
Packed circles showing antenatal care coverage from where pregnant women receive services.

Based on the foregoing the locations where women can be found during pregnancy and the availability or otherwise of PMTCT services are depicted visually in Fig 4.

### Utilization of HIV testing and treatment services by pregnant women [Uptake]

**Fig 5:**
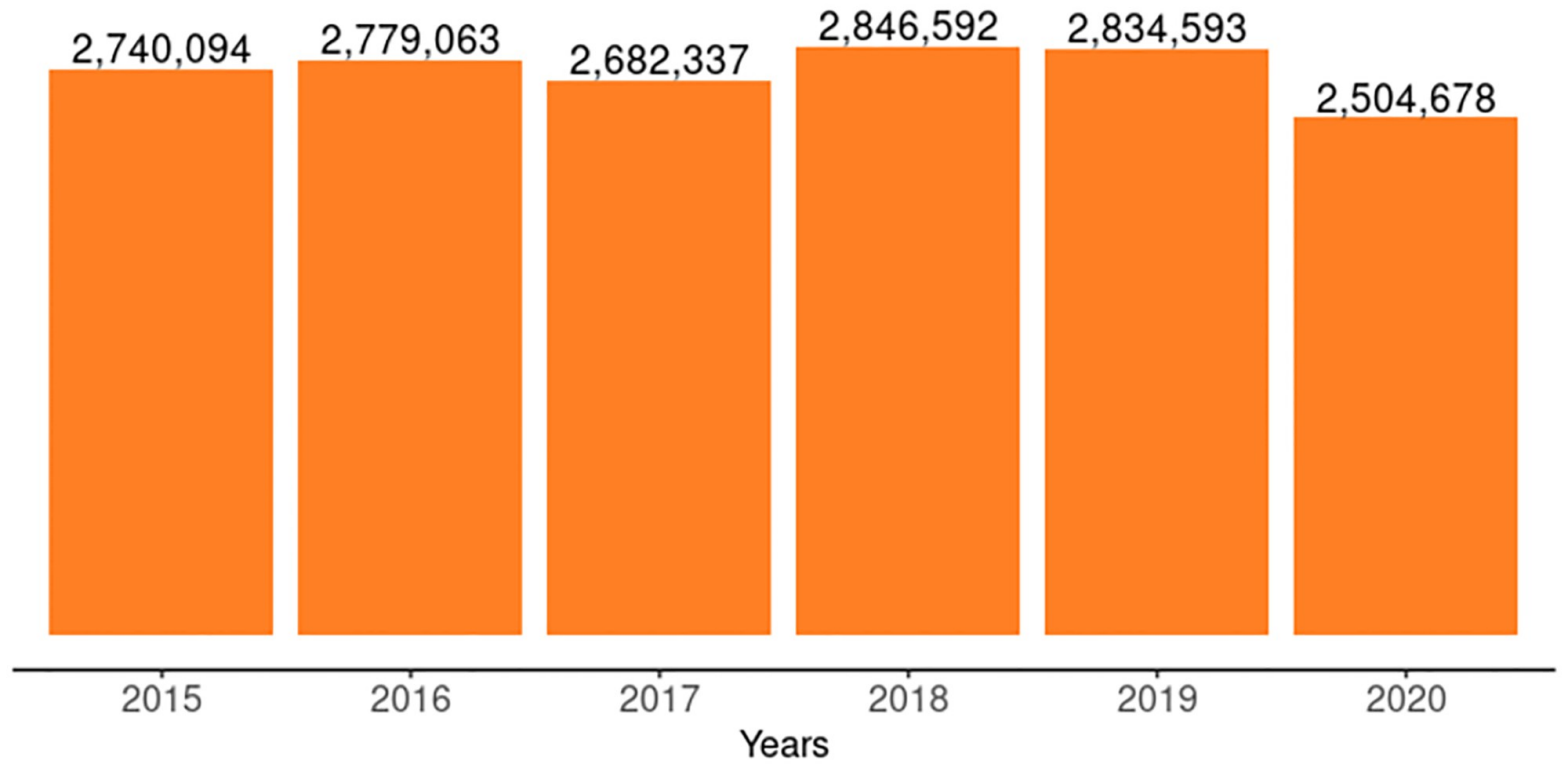
Number of pregnant women tested for HIV and received results annually from 2015–2020.

**Fig 6:**
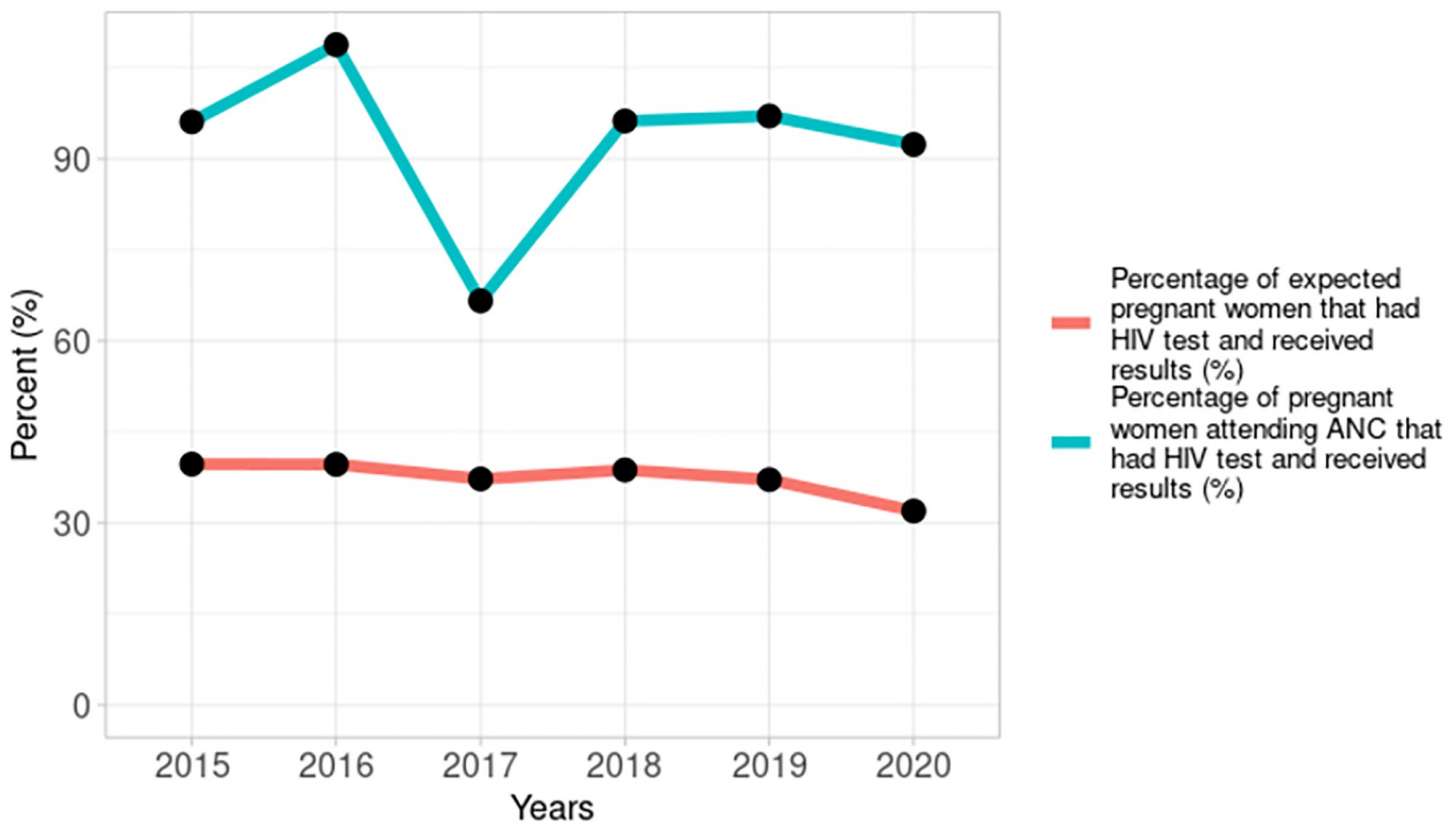
Percentage of pregnant women seen and tested, and percentage of estimated pregnant women tested annually from 2015–2020.

The number of pregnant women tested for HIV annually was fairly constant from 2015 to 2020, as shown in Fig 5, except for 2020 when there was a 12% decline compared to 2019 and there was no trend (tau = -0.0667, 2-sided p value = 1). The testing coverage as a percentage of all estimated pregnant women declined from 40% in 2015 to 32% in 2020. However, among pregnant women who received antenatal care at the PMTCT reporting facilities, testing coverage was above 90% every year except in 2017 when it was 67% (Fig 6).

**Fig 7:**
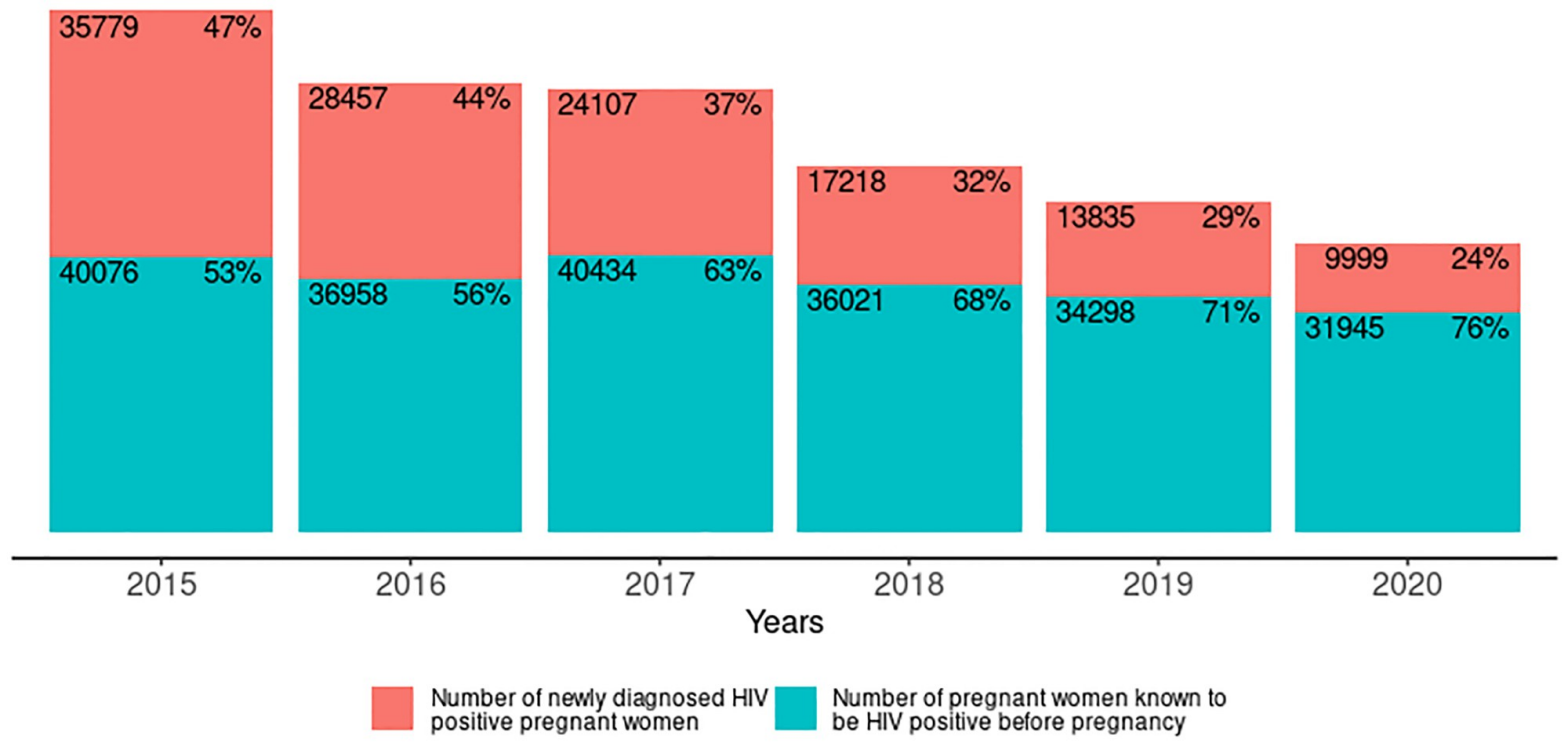
Number of HIV positive pregnant women identified annually disaggregated by new diagnosis and known positive from 2015–2020.

The number of HIV positive pregnant women that received antenatal care at PMTCT reporting facilities declined by 44.7% from 75,855 in 2015 to 41,944 in 2020. The decline in the number of newly diagnosed HIV positive pregnant women was 72.1% compared to 20.3% for pregnant women who already knew that they were HIV positive prior to their first antenatal visit (Fig 7). Annually, the decline in the number of newly diagnosed HIV positive pregnant women ranged from 15–28% compared to 5–11% for women who already knew that they were HIV positive prior to the first antenatal visit.

**Fig 8:**
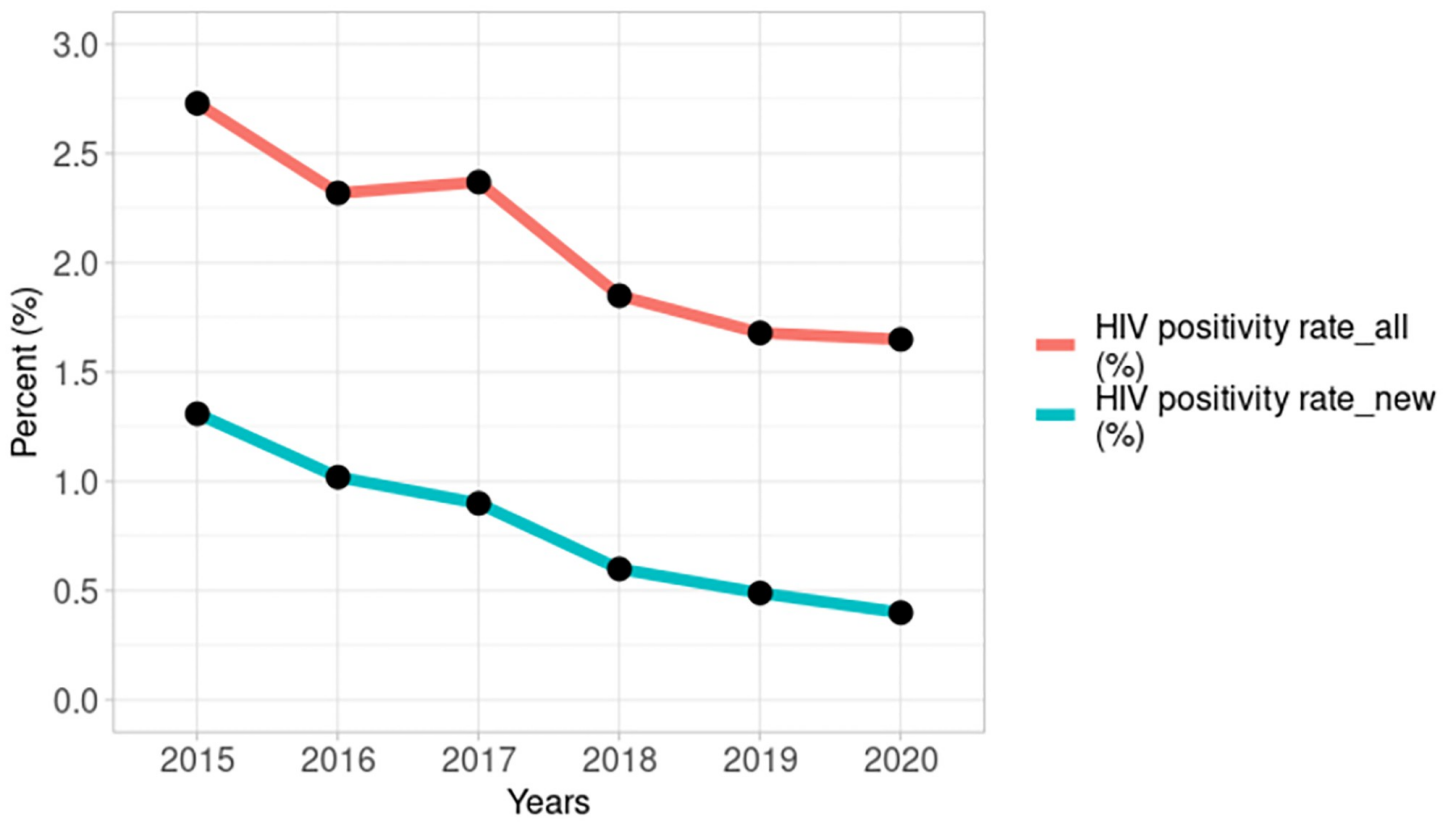
Annual HIV positivity rates among pregnant women considering newly diagnosed HIV positive pregnant women alone and all HIV positive pregnant women from 2015–2020.

The HIV positivity rate when considering only newly diagnosed HIV positive pregnant women declined by 69.5% from 1.31% in 2015 to 0.4% in 2020 (Fig 8). A decline also occurred when all HIV positive pregnant women (newly diagnosed and known positives) were considered but the decline was only by 39.6%.

**Fig 9:**
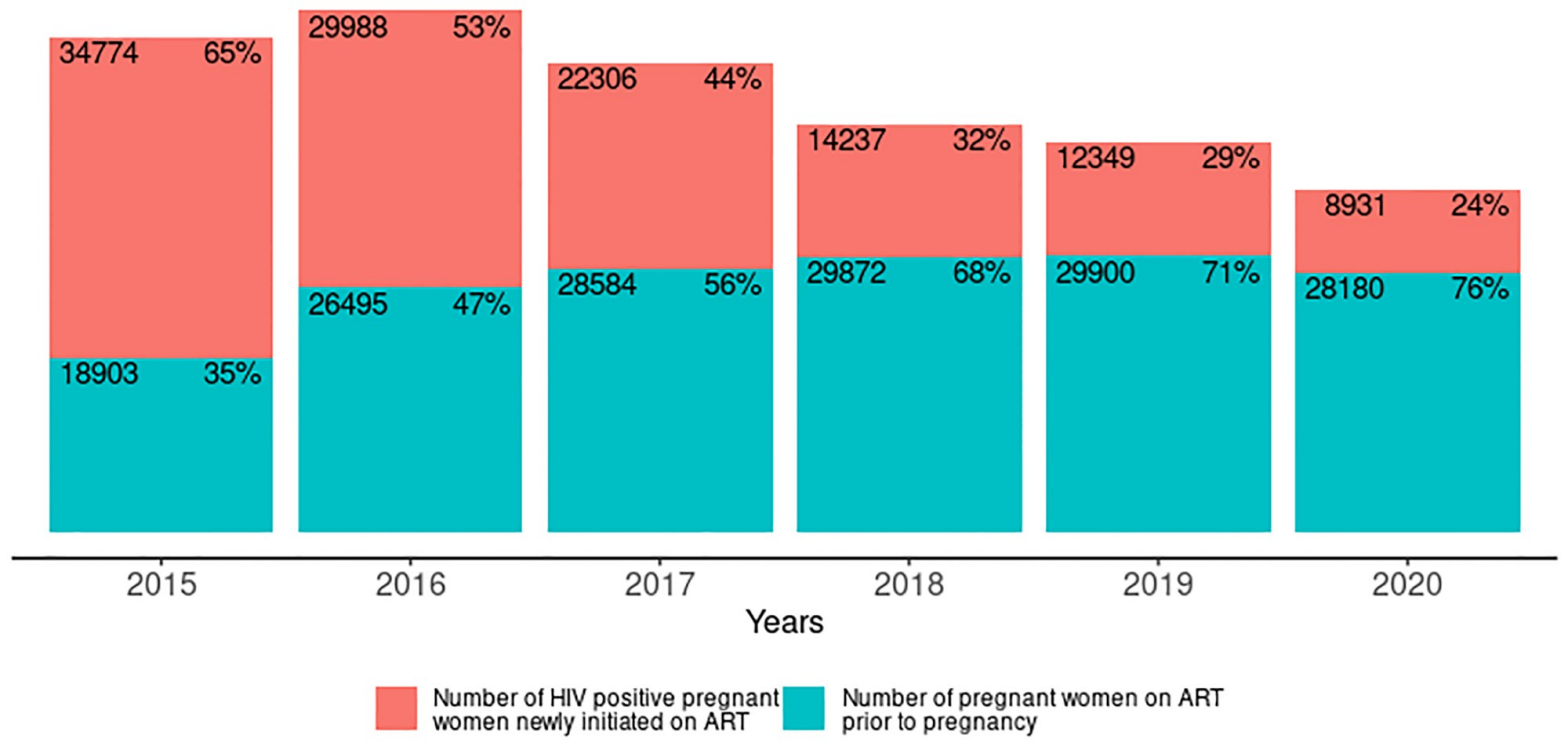
Number of HIV positive pregnant women on antiretroviral treatment annually disaggregated by new initiation on treatment and already on treatment from 2015–2020.

Among pregnant women on ART, the percentage with prior knowledge of their status increased from 35% to 76% between 2015 and 2020 as shown in Fig 9.

**Fig 10:**
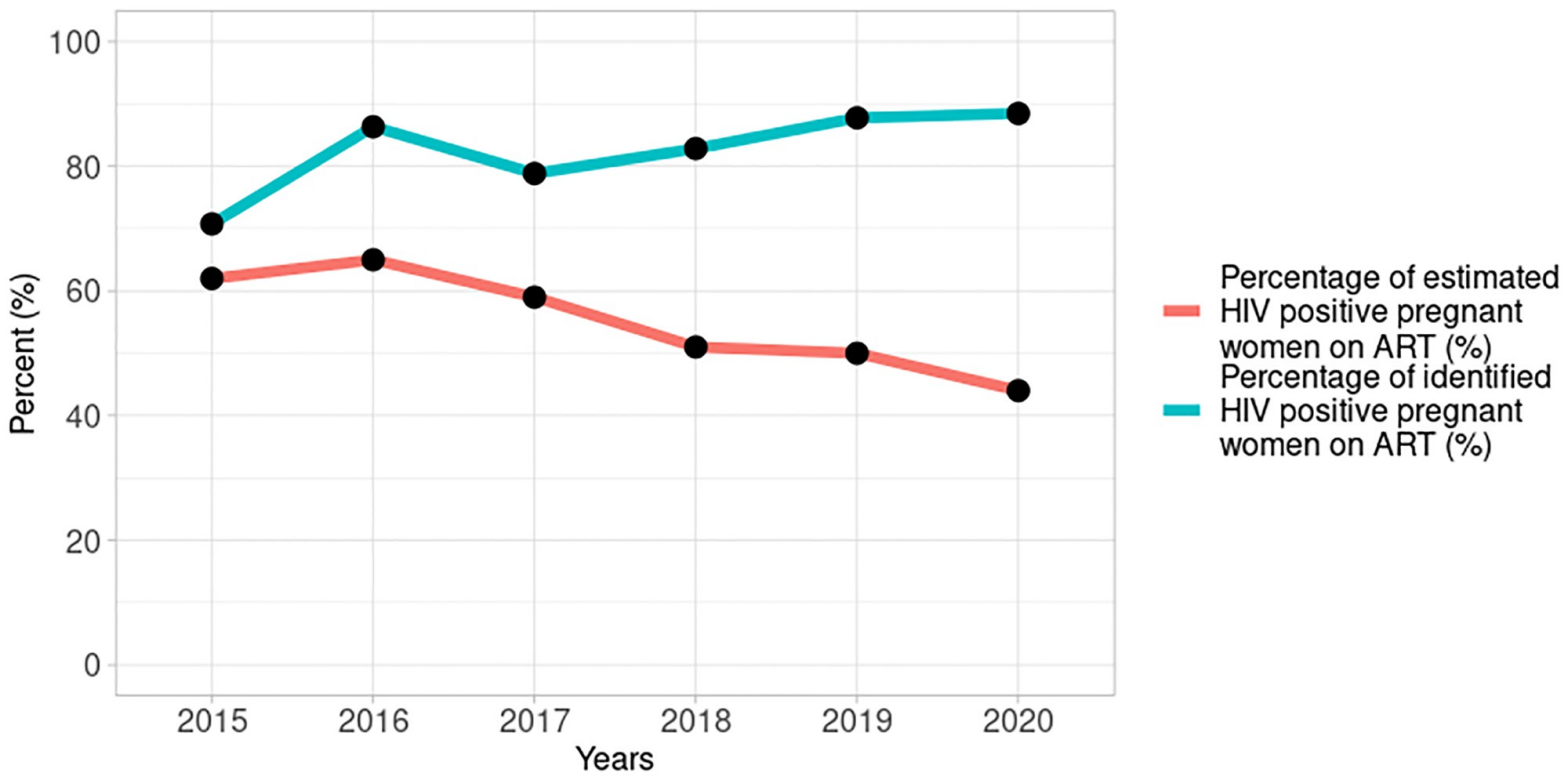
Percentage of estimated and identified HIV positive pregnant women on antiretroviral treatment from 2015–2020.

The progressive decline in the number of HIV positive pregnant women identified and put on treatment in Figs 8 and 9 translates to the declining trend in PMTCT coverage observed in Fig 10 (tau = -0.867, 2-sided p value = 0.024). However, among women identified as HIV positive during pregnancy, the percentage put on ART increased from 71% in 2015 to 88% in 2020.

Mann-Kendall Trend Test statistics for various indicators are shown in Table 2.

**Table 2:** Mann-Kendall trend test statistics for PMTCT indicators.

Indicator	Kendall’s tau statistic	Kendall’s score	Denominator	Variance of Kendall’s score	P-value
Estimated number of total births	1.000	15.000	15.000	28.333	0.009
Number of new antenatal registrations	-0.067	-1.000	15.000	28.333	1.000
Number of pregnant women tested for HIV	-0.067	-1.000	15.000	28.333	1.000
Number of pregnant women known to be HIV positive before pregnancy	-0.733	-11.000	15.000	28.333	0.060
Number of newly diagnosed HIV positive pregnant women	-1.000	-15.000	15.000	28.333	0.009
Number of HIV positive pregnant women identified	-1.000	-15.000	15.000	28.333	0.009
Number of pregnant women on ART prior to pregnancy	0.600	9.000	15.000	28.333	0.133
Number of HIV positive pregnant women newly initiated on ART	-1.000	-15.000	15.000	28.333	0.009
Number of pregnant women on ART	-0.867	-13.000	15.000	28.333	0.024
Number of pregnant women estimated to need ART	-0.602	-7.000	11.619	19.667	0.176
Pregnant women seen at reporting sites as a percentage of estimated number of all pregnant women	-0.467	-7.000	15.000	28.333	0.260
Pregnant women tested at reporting sites as a percentage of estimated number of all pregnant women	-0.828	-12.000	14.491	27.333	0.035
Pregnant women tested as a percentage of pregnant women seen at PMTCT reporting sites efficiency	-0.067	-1.000	15.000	28.333	1.000
Total number of pregnant women seen	-0.067	-1.000	15.000	28.333	1.000
HIV positivity rate among pregnant women tested for HIV	-1.000	-15.000	15.000	28.333	0.009
HIV positivity rate among pregnant women including known positives	-0.828	-12.000	14.491	27.333	0.035
PMTCT coverage	-0.867	-13.000	15.000	28.333	0.024
Pregnant women on ART as a percentage of HIV positive pregnant women identified at PMTCT reporting sites	0.733	11.000	15.000	28.333	0.060

*Green highlighted cells: Statistically significant.

### Differences in PMTCT performance by funder

**Fig 11:**
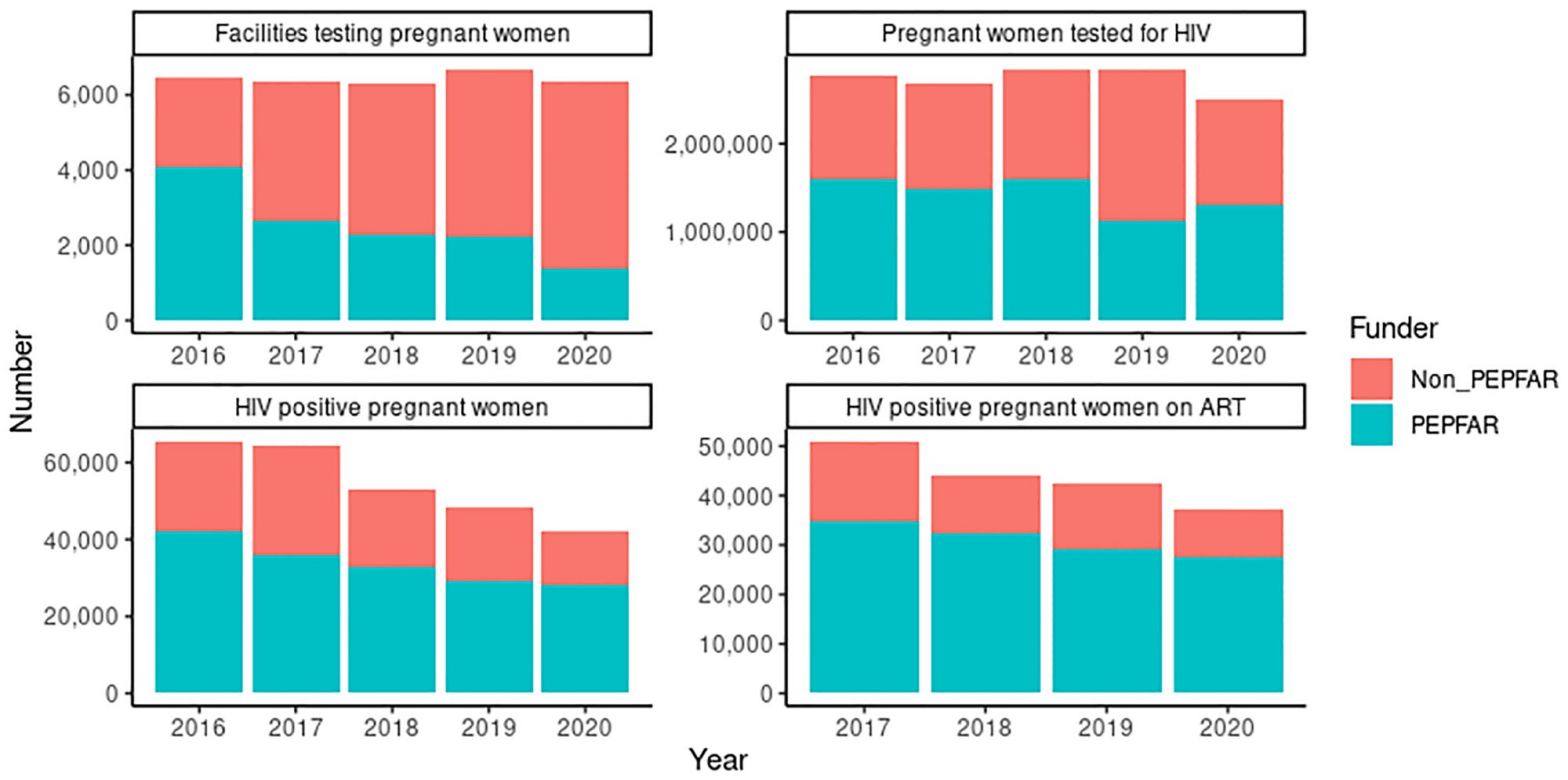
Comparison between PMTCT facilities and services by funder.

**Fig 12:**
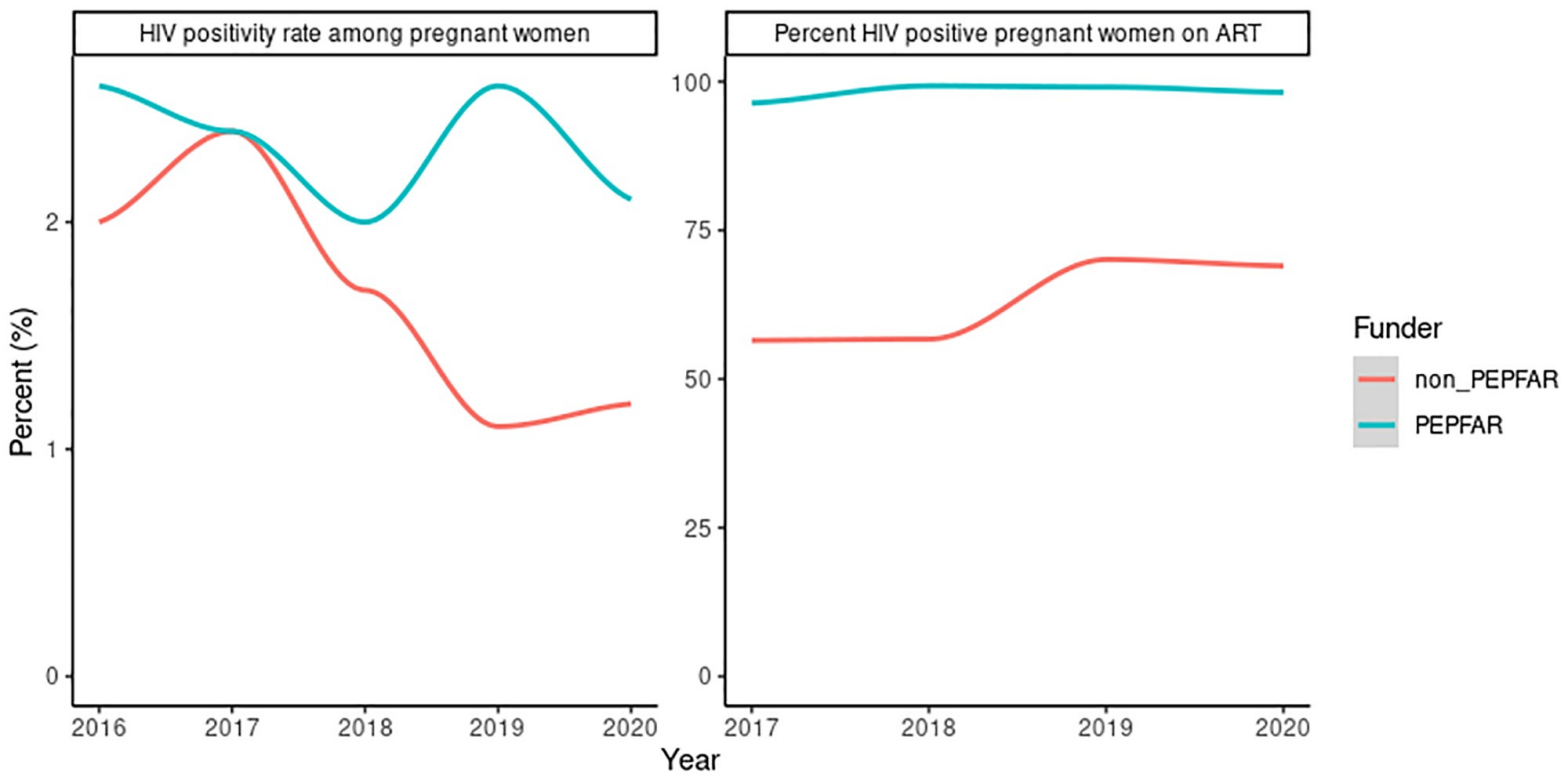
HIV positivity rate and percent of HIV positive pregnant women on ART by funder.

Disaggregation of the data by funder shows important differences. The number of PMTCT sites supported by PEPFAR decreased by 67% from 4,097 in 2016 to 1,357 in 2020 (Fig 11). This is associated with a 18% reduction in the number of pregnant women tested for HIV from 1.60 million in 2016 to 1.23 million in 2020. The number of HIV positive pregnant women identified reduced by 34% from 42,166 in 2016 to 27,948 in 2020. The number of HIV positive pregnant women on ART declined by 21% from 36,176 in 2017 to 27,454 in 2020. The HIV positivity rate was maintained above 2% throughout the period (Fig 12). Among HIV positive pregnant women identified at PEPFAR-supported sites, the percentage on ART ranged from 96% to 99% from 2017 to 2020 (Fig 12).

The number of PMTCT sites supported by other funders (Government of Nigeria, Global Fund, and others) more than doubled from 2,343 in 2016 to 4,976 in 2020 (Fig 11). The number of pregnant women tested for HIV increased from 1,181,920 in 2016 to peak at 1,702,426 in 2019 then declined to 1,303,191 in 2020. The number of HIV positive pregnant women identified peaked at 28,365 in 2017 then declined progressively to 13,996 in 2020. The positivity rate decreased from 1.97% in 2016 to 1.16% in 2020. The number of HIV positive pregnant women on ART decreased from 16,031 in 2017 to 9,657 in 2020. The percentage of HIV positive women on ART increased from 57% in 2017 and 2018 to 70% in 2019 and 69% in 2020 (Fig 12).

## Discussions

To understand Nigeria’s PMTCT performance, we explored two aspects, the low PMTCT coverage and the declining trend.

The low performance of PMTCT in Nigeria is often attributed to low coverage of antenatal care [15–19]. Our findings show a more nuanced situation. Primarily, Nigeria’s PMTCT coverage is low because of limited availability of HIV testing for pregnant women and anti-retroviral treatment for HIV positive pregnant women. We found that only about a third of pregnant women attended ANC facilities which offer and report PMTCT services.

Nigeria’s PMTCT coverage performance over the years has been based on ANC facilities that offer and report on PMTCT services which represent only 25% of ANC facilities. Pregnant women who attend ANC facilities which offer but do not report on the PMTCT services they provide are not counted in the country’s performance measures. Therefore, the reported PMTCT coverage over the years may have been underestimated. There are also pregnant women who receive ANC services at facilities that do not offer PMTCT services. These two groups of women represent about 32% of all pregnant women and 48% of pregnant women who received antenatal care at health facilities.

Improving PMTCT coverage requires an expansion of the number and percentage of antenatal clinics that provide PMTCT services. However, during the period under review, such an expansion was precluded by approaches of implementation that were not aligned to the policy position of universal HIV testing for pregnant women. For instance, based on efficiency of HIV testing from a cost standpoint, there was a major shift to providing selective HIV testing for pregnant women based on pre-defined criteria at facility, local government, and state levels. The criteria for selection of States for scale up of services where ANC clinic attendance of at least 50,000 pregnant women; HIV testing of less than 90% of pregnant women attending ANC clinic; and high positivity yield of positive pregnant women per annum. At local government level, only 32 of the 774 LGAs were selected for scale-up of PMTCT services. At other LGAs, testing coverage was expected to reach no more than 30% of pregnant women. Health facilities were expected to produce a minimum number of positive tests per year. At facilities that did not meet the threshold of at least 5 (and later on at least 12) positive tests among pregnant women per year, there was no demand creation or routine testing for pregnant women at those facilities in subsequent years [16,20–25].

The consequence of these practices is that there has been no expansion in PMTCT coverage in the country. Since the high burden/high volume sites are already providing PMTCT services, any expansion of coverage requires that services are made available at low burden/low volume sites since every positive test counts towards the total number of positive pregnant women identified annually.

The implementation practices described above preclude rapid progress towards eliminating new HIV infections among children and deny women in low burden states and low volume sites access to routine HIV test available to women in high burden, high volume location. This defeats the principle of equity and equal access to PMTCT services and increases inequalities in service uptake [4].

Although performance is low, there has been progressive improvement within the pool of facilities that offer and report on PMTCT numbers. Except for 2020, there was no decline in the number of pregnant women seen and tested at PMTCT reporting sites. Facilities that no longer receive extra support for PMTCT because they do not meet the yield threshold often continue to provide testing services if test kits are available. However, in the absence of the extra support, which usually includes additional personnel, such facilities are not as efficient in ensuring that HIV positive pregnant women identified are put on antiretroviral treatment. Since 2017, PMTCT coverage at PEPFAR supported sites has been 96% or higher compared to a maximum of 70% at non-PEPFAR supported sites. However, at the non-PEPFAR supported sites, the data presented shows that treatment coverage among HIV positive pregnant women has been increasing.

Some qualitative studies suggest that stigma and other factors are significant contributors to low PMTCT coverage [26–29]. However, our findings show that for HIV positive pregnant women identified, there was a progressive increase in the percentage put on antiretroviral drugs reaching 88% in 2020. This suggests that, where services are available, pregnant women accept to be tested for HIV and linked to treatment if positive; possibly, motivated by a desire to prevent transmission of HIV to their infants [30].

So far, we have attempted to explain the low PMTCT coverage in Nigeria. The declining trend in coverage also requires explanation. The expectation was that declining trends in pregnant women tested for HIV contribute to a decline in the number of positive pregnant women identified and put on ART and, thus, a decline in PMTCT coverage. However, despite testing similar numbers of pregnant women each year there has been progressively lower positivity rates. Consequently, fewer positive pregnant women were put on treatment over the period, even when pregnant women who already know their HIV positive status and are on anti-retroviral treatment have been accounted for.

Since the estimated positive pregnant women (denominator) has remained fairly constant from 2015 to 2020, annual reductions in positivity rate translate to annual reductions in PMTCT coverage although treatment coverage among identified HIV positive pregnant women improved annually (as in Fig 10). We postulate that the declining positivity rates may be due to changes in the pool of HIV positive pregnant women who receive care at the PMTCT reporting sites. The NAIIS showed that prevalence of HIV among women increased progressively with age from 0.8% in the 15–24 year age group to 2.0% in the 25–34 year age group and 2.8% among women aged 35–49 years [12]. This could mean that the declining HIV positivity rate among pregnant women may be due to younger women with lower HIV prevalence entering the pregnancy pool and replacing older women with higher HIV prevalence who are leaving the pregnancy pool.

An informed policy change to expand the provision of HIV testing services beyond the 6343 facilities to every service delivery platform has recently been instituted to address the gap in PMTCT services beyond these facilities across the country, to offer HIV testing to all pregnant women, increase case identification and reporting of all services rendered.

We take note of certain variations in the data set which stand out differently. 2017 recorded 4.0 million pregnant women who attended ANC which may be due to a one-off community outreach programme. In 2016, pregnant women tested for HIV as a percentage of pregnant women registered for antenatal care was 109% which can be attributed to testing of pregnant women for HIV before registration for antenatal care. In addition, some pregnant women who were tested for HIV during labour and within 72 hours of birth may not have been registered for birth at the facility where this testing was done. The available data from 2015 is not sufficient to provide disaggregation by PEPFAR supported sites, hence the exclusion from the analysis of differences in PMTCT performance by funder over the period of review. 2020 was quite different from preceding years, presumably because of COVID-19 pandemic. Although comparing the trend analysis of 2015–2020 in the number of pregnant women on ART, testing coverage, positivity rate amongst all positives and PMTCT coverage became non-significant compared to 2015–2019 while number of pregnant women on ART prior to pregnancy became significant, the variations above doesn’t in any way undermine the overall findings.

## Conclusion

The low coverage of PMTCT in Nigeria has been linked to implementation practices aimed at improving cost-efficiency in testing pregnant women for HIV but which served to limit access to PMTCT to about a third of pregnant women annually. The declining performance requires further investigation using nationally representative time series data that is disaggregated by age.

## Recommendations

Ensuring the availability of PMTCT services at all ANC facilities is consistent with the national policy and guidelines which require that all pregnant women receive HIV testing in pregnancy. A first step would be to map all providers of antenatal care whether skilled or unskilled and use this as a basis for organizing the delivery and reporting of PMTCT services.

At present, there is no visibility of the contribution of Traditional Birth Attendants (TBA) and other unskilled providers of antenatal care to testing pregnant women for HIV and providing linkage to antiretroviral therapy. Delivery of PMTCT services through TBAs is an important community-based strategy since they provide antenatal care to 9% of pregnant women]. This strategy has been shown to be feasible and acceptable to both the TBAs and their clients [31]. Achieving elimination of vertical transmission will also require the design and implementation of community-based strategies that support access to and reporting of PMTCT services for the 24% of pregnant women who do not receive any form of antenatal care [10]. Any strategy developed should provide a minimum package of antenatal care services so that PMTCT care is not the only service provided.

## Supporting information

S1 Table Programmatic data on selected indicators across all PMTCT reporting service delivery facility. (XLSX)

S2 Table Programmatic data on selected indicators from PEPFAR PEPFAR-supported and non-PEPFAR supported PMTCT service delivery facility. (XLSX)

S3 Table Mann-Kendall Trend Test statistics for PMTCT indicators. (XLSX)

S4 Table Detailed data for PEPFAR-supported facilities with annualization of the data. (XLSX)

## References

[1] UN General Assembly. Political Declaration on HIV/AIDS: resolution adopted by the General Assembly. A/RES/60/262 [Internet]. 2006. https://data.unaids.org/pub/report/2006/20060615_hlm_politicaldeclaration_ares60262_en.pdf.

[2] UN General Assembly. Political Declaration on HIV and AIDS: Intensifying Our Efforts to Eliminate HIV and AIDS: resolution adopted by the General Assembly. A/RES/65/277 [Internet]. 2011 [cited 2021 Oct 20]. https://files.unaids.org/en/media/unaids/contentassets/documents/document/2011/20110610_UN_A-RES-65-277_en.pdf.

[3] UN General Assembly. Political Declaration on HIV and AIDS: On the Fast-Track to Accelerating the Fight against HIV and to Ending the AIDS Epidemic by 2030: resolution / adopted by the General Assembly. A/RES/70/266 [Internet]. 2016 [cited 2021 Oct 20]. http://onusidalac.org/1/images/2016-political-declaration-HIV-AIDS_en.pdf.

[4] UN General Assembly. Political Declaration on HIV and AIDS: Ending Inequalities and Getting on Track to End AIDS by 2030: resolution / adopted by the General Assembly. A/RES/75/284 [Internet]. 2021. https://digitallibrary.un.org/record/3928975/files/A_RES_75_284-EN.pdf.

[5] UNAIDS. Start Free, Stay Free, AIDS Free—Final report on 2020 targets [Internet]. 2021. https://www.unaids.org/sites/default/files/media_asset/2021_start-free-stay-free-aids-free-final-report-on-2020-targets_en.pdf.

[6] UNAIDS. Confronting Inequalities: Lessons for pandemic responses from 40 years of AIDS. Global AIDS Update 2021 [Internet]. [cited 2021 Oct 30]. https://www.unaids.org/sites/default/files/media_asset/2021-global-aids-update_en.pdf.

[7] UNAIDS. UNAIDS Data 2021. UNAIDS 2021 Reference. https://www.unaids.org/sites/default/files/media_asset/JC3032_AIDS_Data_book_2021_En.pdf.

[8] UNAIDS, Avenir Health. Spectrum 2021, Nigeria File.

[9] National Agency for the Control of AIDS, UNAIDS. Modes of HIV Transmission in Nigeria: Application of the Incidence Patterns Model. 2020.

[10] National Population Commission (NPC) [Nigeria] and ICF. 2019. Nigeria Demographic and Health Survey 2018. Abuja, Nigeria, and Rockville, Maryland, USA: NPC and ICF. [Internet]. https://dhsprogram.com/pubs/pdf/FR359/FR359.pdf.

[11] Federal Ministry of Health Nigeria. Nigeria Health Facility Registry [Internet]. [cited 2021 Oct 30]. http://hfr.health.gov.ng/facilities/hospitals-list.

[12] Federal Ministry of Health, Nigeria. Nigeria HIV/AIDS Indicator and Impact Survey (NAIIS) 2018: Technical Report [Internet]. 2019 [cited 2021 Oct 20]. https://www.naiis.ng/resource/NAIIS-Report-2018.pdf.

[13] amfAR, PEPFAR. PEPFAR Monitoring, Evaluation, and Reporting Database [Internet]. [cited 2021 Oct 30]. https://mer.amfar.org/location/Nigeria.

[14] R Core Team. R: A language and environment for statistical computing. [Internet]. R Foundation for Statistical Computing, Vienna, Austria.; 2021. https://www.R-project.org/.

[15] Olakunde BO, Adeyinka DA, Olawepo JO, Pharr JR, Ozigbu CE, Wakdok S, et al. Towards the elimination of mother-to-child transmission of HIV in Nigeria: a health system perspective of the achievements and challenges. Int Health. 2019 Jul 1;11(4):240–9. doi: 10.1093/inthealth/ihz018 31028402

[16] Country Coordinating Mechanism Nigeria. Nigeria—Funding Request TB/HIV—2020—en [Internet]. [cited 2021 Dec 11]. https://gfdatastore.blob.core.windows.net/files/Applications/Funding%20Requests/NGA/2020/TB_HIV/en/NGA-C_FundingRequest_0_en.zip.

[17] PEPFAR. Nigeria: Country Operational Plan (COP) 2019—Strategic Direction Summary [Internet]. 2019. https://www.state.gov/wp-content/uploads/2019/09/Nigeria_COP19-Strategic-Directional-Summary_public.pdf.

[18] PEPFAR. Nigeria: Country Operational Plan (COP) 2020—Strategic Direction Summary [Internet]. 2020. https://www.state.gov/wp-content/uploads/2020/07/COP-2020-Nigeria-SDS-Final-.pdf.

[19] PEPFAR. Nigeria: Country Operational Plan (COP) 2021—Strategic Direction Summary [Internet]. 2021. https://www.state.gov/wp-content/uploads/2021/09/Nigeria_SDS_Final-Public_Aug-11-2021.pdf.

[20] PEPFAR. Nigeria Country Operational Plan (COP) 2015 Strategic Direction Summary [Internet]. 2015 [cited 2021 Nov 7]. https://www.state.gov/wp-content/uploads/2019/08/Nigeria-14.pdf.

[21] PEPFAR. Nigeria: Country Operational Plan (COP) 2016—Strategic Direction Summary [Internet]. 2016. https://www.state.gov/wp-content/uploads/2019/08/Nigeria-16.pdf.

[22] PEPFAR. Nigeria: Country Operational Plan (COP) 2017—Strategic Direction Summary [Internet]. 2017. https://www.state.gov/wp-content/uploads/2019/08/Nigeria-21.pdf.

[23] PEPFAR. Nigeria: Country Operational Plan (COP) 2018—Strategic Direction Summary [Internet]. 2018. https://www.state.gov/wp-content/uploads/2019/08/Nigeria-2.pdf.

[24] Country Coordinating Mechanism Nigeria. Nigeria—Funding Request TB/HIV—2017—en [Internet]. https://gfdatastore.blob.core.windows.net/files/Applications/Funding%20Requests/NGA/2017/TB_HIV/en/NGA-C_FundingRequest_0_en.zip.

[25] Country Coordinating Mechanism Nigeria. Nigeria—Funding Request HIV 2018—en [Internet]. [cited 2021 Dec 11]. https://gfdatastore.blob.core.windows.net/files/Applications/Funding%20Requests/NGA/2018/HIV/en/NGA-H_FundingRequest_0_en.zip.

[26] Cornelius LJ, Erekaha SC, Okundaye JN, Sam-Agudu NA. A Socio-Ecological Examination of Treatment Access, Uptake and Adherence Issues Encountered By HIV-Positive Women in Rural North-Central Nigeria. J Evid-Inf Soc Work. 2018 Feb;15(1):38–51. doi: 10.1080/23761407.2017.1397580 29236624

[27] Dirisu O, Eluwa G, Adams E, Torpey K, Shittu O, Adebajo S. "I think this is the only challenge… the stigma” Stakeholder perceptions about barriers to Antenatal care (ANC) and Prevention of mother-to-child transmission (PMTCT) uptake in Kano state, Nigeria. PLoS ONE. 2020 Apr 27;15(4):e0232028. 32339180 10.1371/journal.pone.0232028 PMC7185580

[28] Azuogu BN, Ossai EN, Aniwada EC, Madubueze UC, Azuogu VC, Agu AP, et al. Knowledge and Utilization of PMTCT Services among Women accessing Antenatal Care in Private Health Facilities in Abakaliki, Nigeria. West Afr J Med. 2021 Aug 30;38(8):713–8. 34499828

[29] Saka AO, Onyeneho CA, Ndikom CM. Perception and utilization of prevention of mother-to-child transmission of human immunodeficiency virus (HIV) services among women living with HIV. Eur J Midwifery. 2021 Sep 16;5:41. doi: 10.18332/ejm/140454 34604718 PMC8442691

[30] Erekaha SC, Cornelius LJ, Bessaha ML, Ibrahim A, Adeyemo GD, Fadare M, et al. Exploring the acceptability of Option B plus among HIV-positive Nigerian women engaged and not engaged in the prevention of mother-to-child transmission of HIV cascade: a qualitative study. SAHARA J J Soc Asp HIVAIDS Res Alliance. 2018 Sep 25;15(1):128–37. doi: 10.1080/17290376.2018.1527245 30253709 PMC6161587

[31] Nsirim RO, Iyongo JA, Adekugbe O, Ugochuku M. Integration of Traditional Birth Attendants into Prevention of Mother-to-Child Transmission at Primary Health Facilities in Kaduna, North-West Nigeria. J Public Health Afr. 2015 May 13;6(1):455. doi: 10.4081/jphia.2015.455 28299134 PMC5349261

